# Machine learning models for predicting steroid-resistant of nephrotic syndrome

**DOI:** 10.3389/fimmu.2023.1090241

**Published:** 2023-01-26

**Authors:** Qing Ye, Yuzhou Li, Huihui Liu, Jianhua Mao, Hangjin Jiang

**Affiliations:** ^1^ Department of Clinical Laboratory, The Children’s Hospital, Zhejiang University School of Medicine, National Clinical Research Center for Child Health, National Children’s Regional Medical Center, Hangzhou, China; ^2^ Center for Data Science, Zhejiang University, Hangzhou, China; ^3^ School of Mathematical Sciences, Zhejiang University, Hangzhou, China; ^4^ Department of Nephrology, The Children’s Hospital, Zhejiang University School of Medicine, National Clinical Research Center for Child Health, National Children’s Regional Medical Center, Hangzhou, China

**Keywords:** idiopathic nephrotic syndrome, steroid responsiveness, machine learning, prediction model, nephrotic syndrome

## Abstract

**Background:**

In the absence of effective measures to predict steroid responsiveness, patients with nonhereditary steroid-resistant nephrotic syndrome (SRNS) have a significantly increased risk of progression to end-stage renal disease. In view of the poor outcomes of SRNS, it is urgent to identify the steroid responsiveness of idiopathic nephrotic syndrome (INS) early.

**Methods:**

To build a prediction model for SRNS, we collected 91 subjects; 57 of them had steroid-sensitive nephrotic syndrome, and the others had SRNS. For each subject, 87 clinical variables were measured. In general, only a small part of these variables is informative to SRNS. Thus, we proposed a new variable selection framework including a penalized regression approach (named MLR+TLP) to select variables having a linear effect on the SRNS and a nonparametric screening method (MAC) to select variables having a nonlinear marginal (joint) effect on the SRNS. Thereafter, considering the correlation between selected clinical variables, we used a stepwise method to build our final model for predicting SRNS. In addition, a statistical testing procedure is proposed to test the overfitting of the proposed model.

**Results:**

Twenty-six clinical variables were selected to be informative to SRNS, and an SVM model was built to predict SRNS with a leave-one-out cross-validation (LOO-CV) accuracy of 95.2% (overfitting p value<0.005). To make the model more useful, we incorporate prior medical information into the model and consider the correlation between selected variables. Then, a reduced SVM model including only eight clinical variables (erythrocyte sedimentation rate, urine occult blood, percentage of neutrophils, immunoglobulin A, cholesterol, vinculin autoantibody, aspartate aminotransferase, and prolonged prothrombin time) was built to have a LOO-CV accuracy of 92.8% (overfitting p value<0.005). The validation cohort showed that the reduced model obtained an accuracy of 94.0% (overfitting p value<0.005), with a sensitivity of 90.0% and a specificity of 96.7%. Notably, vinculin autoantibody is the only podocyte autoantibody included in this model. It is linearly related to steroid responsiveness. Finally, our model is freely available as a user-friendly web tool at https://datalinkx.shinyapps.io/srns/.

**Conclusion:**

The SRNS prediction model constructed in this study comprehensively and objectively evaluates the internal conditions and disease status of INS patients and will provide scientific guidance for selecting treatment methods for children with nonhereditary SRNS.

## Introduction

Idiopathic nephrotic syndrome (INS) is the most common glomerular disease in children and is characterized by proteinuria, hypoproteinemia, and edema (1, 2). Glucocorticoids (GCs) are the first-line treatment for INS. It can induce remission in approximately 80% of children and is known as steroid-sensitive nephrotic syndrome (SSNS). However, 10% - 20% of children still have steroid-resistant nephrotic syndrome (SRNS) and need to be supplemented with immunosuppressants (3). Children with SSNS have a good renal prognosis. The risk of developing chronic kidney disease (CKD) in SSNS patients is estimated to be less than 5% ten years after diagnosis (4). In contrast, children with SRNS have a significantly increased risk of progression to end-stage recurrent disease (ESRD) (5). Children with SRNS on biopsy had focal segmental glomerulosclerosis (FSGS), and 50% of them had a risk of progression to ESRD within five years (4, 6). It has been reported that SRNS is the second most common cause of CKD in the first 30 years of life (7).

At present, many studies have found that approximately one-third of children with SRNS have a genetic background. Dysfunction of the glomerular filtration barrier (GFB) is the main pathological mechanism of SRNS. The loss of its normal selective permeability leads to proteinuria (8). To date, more than 60 gene mutations related to GFB function have been found in children with SRNS, such as *NPHS1, NPHS2*, and *MYOIE*. Deletion of these genes in the human body causes GFB function defects (9–11). Unfortunately, in the absence of effective measures to predict steroid responsiveness, approximately 70% of patients with nonhereditary SRNS are at high risk of side effects and disease progression due to prolonged ineffective GCs treatment.

Notably, immune factors also play an important role in the pathogenesis of INS. An increasing number of researchers have found that the potential role of B cells in INS is under discussion due to the therapeutic effect of anti-CD20 antibodies and the identification of pathogenic antibodies against podocyte-expressed proteins, in addition to T lymphocyte dysfunction or dysfunction (12, 13). In INS children, the antibody specifically binds to the target antigen on podocytes, which interferes with the normal function of GFB and causes proteinuria. In our previous study, at least 66% of INS children had podocyte autoantibodies. These podocyte autoantibodies were positively correlated with proteinuria, and their titers decreased rapidly after effective treatment (14, 15). This suggests that the level of podocyte autoantibodies may be a good biomarker for predicting steroid responsiveness. Considering the poor outcomes of SRNS, early identification of the steroid responsiveness of INS is urgent.

This study used 78 laboratory parameters and podocyte autoantibodies to predict steroid responsiveness. To build a precise and efficient model, we proposed a new variable selection procedure that includes an SVM-based and model-free variable selection procedure. The SVM-based variable selection procedure is a model-based method that tends to select variables (marginally or partially) informative to the response according to the model. However, the model-free variable selection procedure tends to select variables having a nonlinear marginal (pairwise joint) effect on the response without any assumptions about the model. Thus, these two subprocedures tend to complement each other in real applications. Applying this new variable selection procedure to this study gives 26 important variables and an SRNS prediction model with a leave-one-out cross-validation (LOO-CV) accuracy of 95.2% (overfitting p value< 0.005). Note that we also propose a statistical test method for testing the overfitting of a statistical (machine learning) model. Although this full model is promising, it still contains too many clinical variables. Taking into consideration the correlation between selected variables, we used a stepwise strategy to build a model only including erythrocyte sedimentation rate (ESR), urine occult blood (u-OB), percentage of neutrophils (N%), IgA, cholesterol (CHOL), vinculin autoantibody, aspartate aminotransferase (AST) and prolonged prothrombin time (PT), which has a LOO-CV accuracy of 92.8%, very close to the full model (overfitting p value<0.005).

## Methods

### Selection of subjects

A total of 91 subjects were recruited at the Children’s Hospital, Zhejiang University School of Medicine, between September 2020 and September 2021. All enrolled patients met the International Study of Kidney Disease in Children (ISKDC) criteria for INS. Patients with suspected heritable nephrotic syndrome, reduced renal function, infectious diseases, malignant tumors, or other autoimmunological diseases were excluded. Children who respond well to steroids within four weeks are considered to have SSNS. Otherwise, it is considered an SRNS. The subjects in this study were divided into two groups: one group included 34 patients with SRNS, and another group included 57 patients with SSNS. Patients with SSNS received steroid treatment of 2 mg/(kg**–**d) for four weeks, whereas for refractory patients, tacrolimus was added at 0.05-0.15 mg/(kg**–**d).

### Data collection

Blood and urine samples were collected from each subject after INS diagnosis and before steroid treatment. A total of 87 variables were collected (**Supplementary Table 1**). Demographic characteristics were collected, including age, sex, and weight. By hematological tests, 43 variables were analyzed, including white blood cell counts, percentage of neutrophils, percentage of lymphocytes, hemoglobin, platelet, C-reactive protein, ESR, total protein, albumin, globulin, alanine aminotransferase, aspartate aminotransferase, serum creatinine, urea, serum cystatin c, serum β2-MG, triglyceride, cholesterol, antistreptococcal hemolysin O, prolonged prothrombin time, fibrinogen, prolonged activated partial thromboplastin time, prolonged thrombin time, D-dimer, IgG, IgA, IgM, C3, C4, retinol conjugated protein, total IgE, IL-2, IL-4, IL-6, IL-10, TNF, IFN-γ, CD19%, CD3%, CD4%, CD8%, CD3^-^CD16^+^CD56^+^%, and CD4/CD8. By urine tests, 25 variables were analyzed, including urine occult blood, urine protein, urine specific gravity, urinary RBC, urinary WBC, urinary microprotein, 24-hour urine protein, urinary microalbumin, urinary α1-MG, urinary β2-MG, urinary transferrin, urinary retinol conjugated protein, urinary IgG, uric acid, 24-hour uric acid, urinary protein/creatinine, urinary calcium, 24-hour urinary calcium, urinary calcium/creatinine, urinary microalbumin/creatinine, urinary α1-MG/creatinine, urinary β2-MG/creatinine, urinary transferrin/creatinine, urinary retinol conjugated protein/creatinine, and urinary IgG/creatinine. A total of 17 autoantibodies to podocyte proteins were detected, including talin-1 (Tln1), moesin (Msn), myosin light chain 1 (Myh1), vinculin (Vcl), aconitate hydratase, mitochondrial (Aco2), cytoskeleton-associated protein 4 (Ckap4), desmoglein 1 (Dsg1), proteasome subunit alpha type-1 (Psma1), F-actin-capping protein subunit beta (Capzb), filamin-A (Flna), plectin (Plec), heat shock protein HSP 90-beta (Hs90a), peptidyl-prolyl cis-trans isomerase D (Ppid), peroxiredoxin-1 (Prdx1), alpha-enolase (Eno1), neuroblast differentiation-associated protein AHNAK (Ahnak), and serine/arginine-rich splicing factor 9 (Sfrs).

### Detection of podocyte autoantibodies

According to a previous method (14), the autoantigens were spotted on a nitrocellulose membrane (0.8 μm pore size, manufactured by Sartorius, Germany) using a chip sampling apparatus (model: AD1500, manufactured by BioDot), and biotin-labeled mouse anti-human immunoglobulin G by Thermo Fisher and 56°C-inactivated serum were also spotted onto the nitrocellulose membrane as positive and negative controls, respectively. The nitrocellulose membrane spotted by autoantigens was soaked in 5% bovine serum albumin sealing solution for one h (the buffer system was Tween-Tris-buffered saline with a pH of 7.4.) and then dried in the oven. The nitrocellulose membrane was then fixed in the groove of a polyvinyl chloride assay plate. Then, 300 μL of patient serum was added to the groove of the assay plate. After incubation and washing with Tris buffer five times, we added 300 μL of biotin anti-human IgG antibody complex to the assay plate. After incubation and washing, we washed the assay plate with running water and read its optical density value with a scanner.

### Data preprocessing

Summary statistics, such as the maximum, minimum, mean, etc., of these 87 clinical variables are given in **Supplementary Table 2**. Continuous variables are normalized to have mean 0 and variance 1. Additionally, variables with too many missing values (missing rate > 50%) are deleted. Finally, 78 clinical variables were used for downstream analysis.

### SVM-based variable selection

Let *n*=91  be the sample size in this study, *X*_*i*_=(1, *x*_*i*1_, *x*_*i*2_, …., *x*_*i*, 87_) be the observations of the 87 clinical variables measured for the *i* -th subject plus the intercept term, and *y*_*i*_ be the corresponding response, SSNS or SRNS, where *i*=1,2,…, *n*. We aim to build an SVM model using part of these 87 clinical variables to predict SSNS or SRNS. To introduce our SVM model, we first define the hinge loss function as $L_{H}\left(\beta \right)=n^{-1}\sum_{i=1}^{n}{\left(1-y_{i}X_{i}^{T}\beta \right)}_{+}$ , where (*z*)_+_=max(0, *z*) . Then, building an SVM model minimizes *L*_*H*_(*β*)+*λ*|*β*|^2^ , which is in the same spirit as ridge regression built upon squared loss instead of hinge loss, and the term *P*_*λ*_(|*β*|)=*λ*|*β*|^2^ is called ridge regularization (or ridge penalty). The aim of variable selection cannot be attained by ridge regularization (16). Well-known penalties designed for variable selections are LASSO (17), SCAD (16), MCP (18), and TLP (19), whose definitions are given below.


$$\text{SCAD: }P_{\lambda }\left(\left|\;\beta \right|\right)=\{\begin{array}{c} \lambda \left|\;\beta \right|,\;\;\;\;\;\;\;\;\;\;if\left|\;\beta \right|\leq \lambda ,\\ \frac{a\lambda \left|\;\beta \right|-\left(\lambda^{2}+\;\beta^{2}\right)/2}{a-1},\;if\lambda \leq \left|\;\beta \right|\leq a\lambda ,\\ \frac{a\lambda^{2}}{2},\;\;\;\;\;\;\;\;\;\;\;\;if\left|\;\beta \right|>a\lambda . \end{array}\;\;\;(a>0)$$

$$\text{MCP:}P_{\lambda }\left(\left|\;\beta \right|\right)=\{\begin{array}{c} \lambda \left(\left|\;\beta \right|-\frac{\theta^{2}}{2a\lambda }\right),\;if\left|\;\beta \right|\leq a\lambda ,\\ \;\;\;\;\;\;\;\;\frac{a\lambda^{2}}{2},\;\;\;\;\;\;\;\;\;\;\;\;if\left|\;\beta \right|>a\lambda . \end{array}\;(a>0)$$

LASSO: *P*_*λ*_(|*β*|)=*λ*|*β*|


$$\text{TLP:}P_{\lambda }\left(\left|\;\beta \right|\right)=TLP\left(\left|\;\beta \right|,\tau \right)\lambda =\min\left(\frac{\left|\;\beta \right|}{\tau },1\right)\lambda \;\;\;\;\;(\tau >0)$$

It is shown that the LASSO penalty introduces bias into parameter estimation, and SCAD and MCP tend to select more irrelevant variables than TLP (19). Thus, in this paper, we take the TLP as the penalty function to achieve variable selection; that is, we consider building an SVM model by solving the following problem: minimize


$$L_{H}\left(\beta \right)+\sum_{i=1}^{n}\min\left(\frac{\left|\;\beta \right|}{\tau },1\right)\lambda \;$$

However, it is difficult to solve this optimization problem due to the discontinuity of the derivative of the hinge loss *L*_*H*_(*β*). Thus, we use a modified logistic regression (20) (MLR) function to approximate the hinge loss, which is defined as


$$L_{\gamma }\left(\beta \right)=\frac{1}{n\gamma }\sum_{i=1}^{n}\text{log}\left(1+e^{\gamma \left(1-y_{i}\left(x_{i}^{T}\beta \right)\right)}\right)$$

It is easy to see that *L*_*γ*_(*β*) → *L*_*H*_(*β*) , as *γ* → *∞* .Thus, we build the SVM model by minimizing *L*_*γ*_(*β*) with the TLP penalty,


$$\frac{1}{n\gamma }\sum_{i=1}^{n}\text{log}\left(1+e^{\gamma \left(1-y_{i}\left(x_{i}^{T}\beta \right)\right)}\right)+\min\left(\frac{\left|\beta^{i}\right|}{\tau },1\right)\lambda$$

and denote this method as MLR+TLP.

### Model-free variable selection

Variable selection based on SVM only selects variables having a linear effect on the response. However, some informative variables have a nonlinear effect on the response. Thus, we use a recently proposed nonparametric variable screening method, MAC (21), to select variables having nonlinear marginal (MAC1) and joint effects (MAC2) on the response. There are two types of joint effects for each pair of variables: T1) both have no marginal effect on the response, and T2) only one has a marginal effect on the response. In summary, we first use MAC1 to select variables with marginal effects and then use MAC2 to select variable pairs with joint effects.

### Model training

As previously mentioned, there are three different classes of variables selected by our method: (1) variables selected based on SVM; we model each variable X in this class as linear, i.e., *βX* .(2) variables selected by MAC due to their marginal effect; these variables are modeled using B-splines denoted by *B*_*s*_(*X*), and the order *s* of B-splines are selected under the control of overfitting; and (3) variables selected by MAC due to their joint effect. These variables are modeled similarly to those in class (2). Finally, the model is trained and tested using leave-one-out cross-validation due to this study’s relatively small sample size. Missing data problems occurred in our study. Although data imputation makes use of partially observed samples, it induces unknown bias in the analysis. Thus, subjects with missing values on selected variables are not included in model training and testing.

### Statistical test for overfitting

It is crucial to eliminate overfitting for machine learning models for their application to other datasets. Here, we propose a statistical test procedure for overfitting with the null hypothesis that the model is overfitted. The principle of the procedure is that given explanatory variable X, a model *f*(*θ*, *X*) is considered to be overfitting if its accuracy of predicting the real response *Y* is not significantly higher than that of predicting a random and independent response. Let *T*_0_ be the true accuracy of model *f*(*θ*, *X*) in predicting the true response. Next, we obtain the performance of the model in predicting a random response. Taking b=1, 2, …, B, for each b, we (1) generate a random response *Y*_*b*_ by permuting the true response Y (2), train the model *f*(*θ*, *X*) using *Y*_*b*_ , and (3) obtain the random accuracy. *T*_*b*_ for predicting *Y*_*b*_ . Now, we have B random accuracies. In other words, we obtain the distribution of the accuracy of predicting a random response. If model *f*(*θ*, *X*) is not overfitting, the true accuracy *T*_0_ should be significantly larger than the random accuracy, that is,. ould be located at the right tail of the distribution. Thus, we define the p value for the overfitting test as p value = $\frac{\sum_{b=1}^{B}(T_{b}>T_{0}\;)+1}{B+1}$ , and a p value<0.05 means we should reject the null hypothesis, i.e., the model is not overfitted. In our analysis, we take B=200.

### Statistical analysis

All statistical analyses were performed using R 4.1.1. P values< 0.05 were considered to indicate statistical significance in statistical tests. In this study, we use the leave-one-out cross-validation (LOO-CV) accuracy and the following measurements to show the performance of different models.


$$\text{SN}=\frac{\text{TP}}{\text{TP}+\text{FN}}$$

$$\text{SP}=\frac{\text{TN}}{\text{TN}+\text{FP}}$$

$$\text{Precision}=\frac{\text{TP}}{\text{TP}+\text{FP}}$$

$$\text{ACC}=\frac{\text{TP}+\text{TN}}{\text{TP}+\text{FN}+\text{TN}+\text{FP}}$$

$$\text{MCC}=\frac{\text{TP}\times \text{TN}-\text{FP}\times \text{FN}}{\sqrt{\left(\text{TP}+\text{FP}\right)\left(\text{TP}+\text{FN}\right)\left(\text{TN}+\text{FP}\right)\left(\text{TN}+\text{FN}\right)}}$$

where TP is the number of true positives, FN is the number of false negatives, FP is the number of false positives, and TN is the number of true negatives.

## Results

### Data characteristics and the analysis workflow

**Figure 1:**
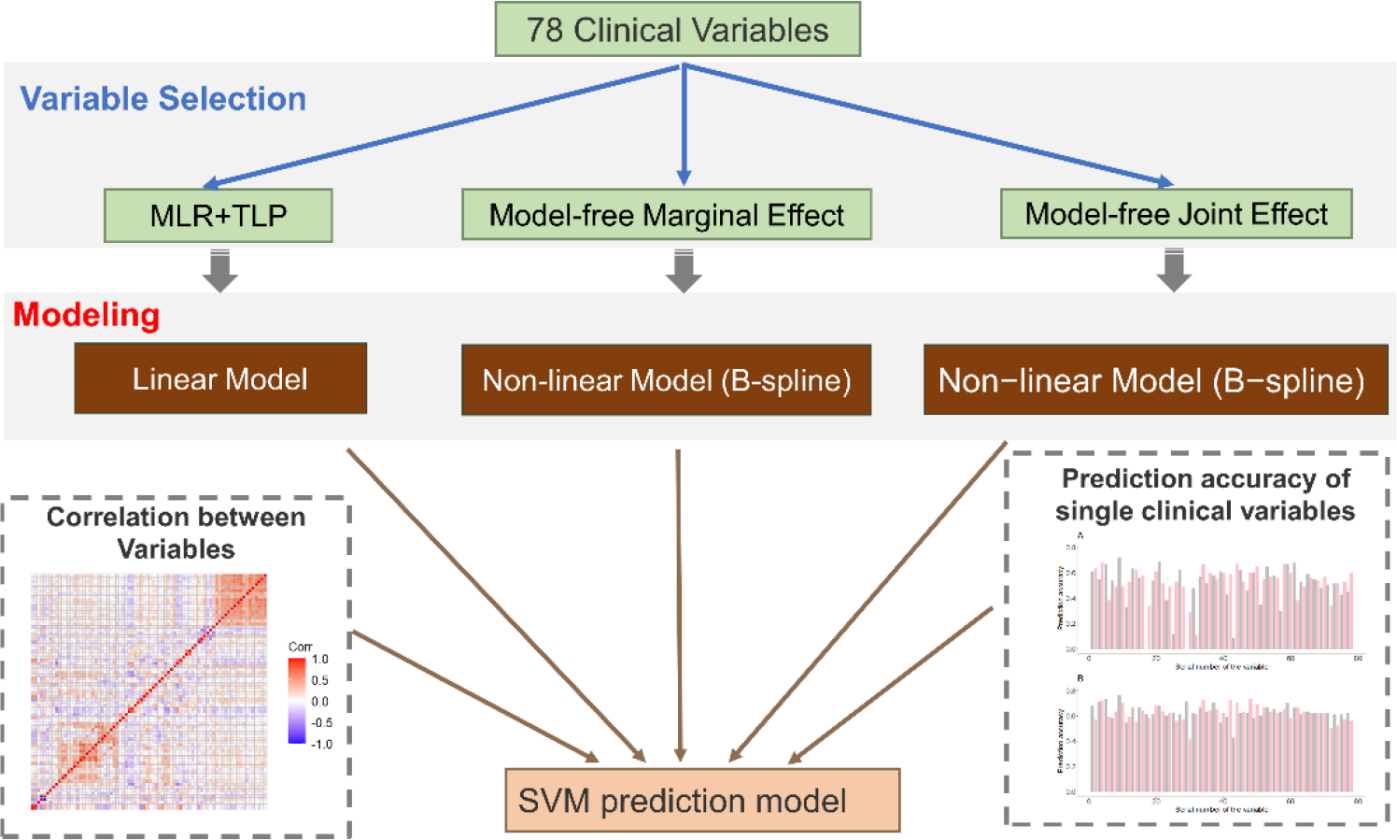
Overview of the method. First, we propose a new variable selection procedure to select informative variables. This procedure contains two parts: SVM-based variable selection (MLR+TLP) and model-free variable selection (MAC). Then, by considering the correlation between selected variables, we use a stepwise regression strategy to build our final prediction model.

A total of 91 newly diagnosed pretreatment subjects comprising 57 patients with SSNS and 34 patients with SRNS were enrolled in the present study. The dataset consisted of 78 variables covering demographic, hematological, and urinary characteristics and podocyte antibodies. The distribution characteristics of each variable are summarized in **Supplementary Table 2**. To analyze the above data, we developed a novel variable selection procedure to select informative and meaningful variables (**Figure 1**). The essence of the approach is to build our machine learning models after a careful variable selection procedure. The overall workflow of the variable selection procedure and the prediction model is as follows.

**Figure 2:**
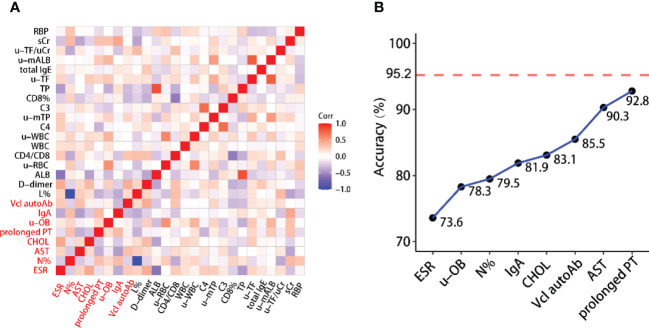
**(A)** Heatmap of correlations between selected variables. This shows that the variables contained in the reduced model have weak correlations. **(B)** Building the reduced model is based on selected variables while considering the correlation between them. The reported accuracy is the LOO-CV accuracy.

First, we use two different methods to select informative variables: SVM-based variable selection (MLR+TLP) and model-free variable selection (MAC) (see Methods for details). MLR+TLP is a model-based variable selection method that tends to select variables with a linear effect on the response, but MAC tends to select variables with a nonlinear effect on the response. Then, SVM models are trained and tested based on selected variables. However, the clinical variables in this study showed strong correlations (**Figures 1** and **2A**); thus, we introduced a greedy pruning stage to prune the prediction model by considering the correlation between the selected variables. Finally, a machine learning model is built after a careful variable selection procedure. The details of variable selection to training the models are described in the following sections.

### Selection of variables with a linear effect on SRNS

**Figure 3:**
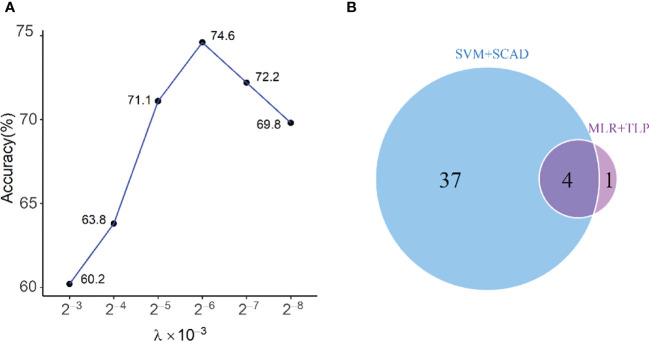
**(A)** Selecting λ in MLR+TLP using leave-one-out cross-validation; **(B)** Venn diagram shows the relationship between chosen variables by MLR+TLP and SVM+SCAD. The four variables selected by the two methods are L%, ALB, N%, and C4, and the one elected only by MLR+TLP is Vcl, which is very important.


In this part, variables with a linear effect on the response were selected by SVM-based variable selection (MLR+TLP). There are two tuning parameters in our model, τ and λ. Following (21, 22), we set τ = 0.0001 and select λ by cross-validation from the range (1 ∼ 2^−10^)×10^−3^ (**Figure 3A**). This method gives five clinical variables: L%, N%, ALB, C4, and vinculin autoantibody, with a LOO-CV accuracy of 74.6% and an overfitting p value< 0.005. The predictive model is given by

sign(0.2527−0.4493×L%−0.7948×N%−0.2683×ALB+0.2879×C4+0.0925×Vcl)

As a comparison, we also run the SVM model with the SCAD penalty (SVM+SCAD) on this dataset, which selects 41 clinical variables with a LOO-CV accuracy of 80.2%. The relationship between variables selected by MLR+TLP and SVM+SCAD is given in **Figure 3B**. Compared with MLR+TLP, the results from SVM+SCAD are not good for selecting too many variables but have a comparative accuracy with that from MLR+TLP. Furthermore, the overfitting p value (=0.031) of the model from SVM+SCAD shows its potential risk of overfitting.

Overall, L%, N%, ALB, C4, and vinculin autoantibodies were singled out, with a linear effect on the response. The levels of L%, N%, and ALB are negatively related to SRNS. The levels of C4 and vinculin autoantibodies are positively related to SRNS. INS patients generally suffer from hypoalbuminemia and high C4 levels. It is suggested that the more severe the disease for INS patients, the more likely it is to be resistant to steroids. The occurrence of vinculin autoantibodies in INS patients demonstrates the activation of B cells and damage to the podocyte actin cytoskeleton. Higher vinculin autoantibody levels are less likely to be sensitive to steroids, and treatment with B-cell-depleting anti-CD20 antibodies may be helpful for these patients.

### Selection of variables with a nonlinear effect on SRNS

Now, the model-free variable selection selects variables with a nonlinear effect on the response. First, we used MAC1 to select variables with a marginal main effect on SRNS, and we obtained 26 clinical variables with a p value<0.05 (**Supplementary Table 3**). Among them, L%, N%, ALB, and C4 were selected again, but vinculin autoantibodies were missed. This is consistent with the fact that MAC1 tends to select marginal main effects but may lose variables showing a strong partial effect (21). Second, MAC2 is used to select variables with joint effects on the response to identify the interaction between multiple variables. It selects hundreds of pairs of joint effects (**Supplementary Tables 4**, **5**).

Although 26 variables are selected by MAC1, many variables are challenging to analyze. Meanwhile, hundreds of pairs of joint effects selected by MAC2 also exhibit similar problems. Therefore, the variable pairs having joint effects on the response are ignored in the downstream analysis.

### Prediction models

We used the 27 clinical variables selected by MAC1 and MLR+TLP to build an SVM for predicting SRNS and obtained a LOO-CV accuracy of 95.2% (overfitting *p* value<0.005). This model is called “the full model” for convenience. The performance of the full model is promising, but it contains too many variables for clinical applications.

We explored the internal relationship between 27 variables to further optimize the model. **Figure 2A** shows that there is a strong correlation between the selected variables. Assuming that the current model includes variable X, it is known that adding a variable W strongly correlated to X does not help much to improve the model’s performance but increases the model complexity. This allows us to reduce the complexity of the full model without losing much accuracy by removing some correlated variables. Motivated by this fact, we use a stepwise forward regression method to build a reduced model with relatively low accuracy. To this aim, we start from a one-variable SVM model with the best performance and add another variable from the remaining 25 variables that give the best performance (**Figure 2B**). Finally, we obtained a reduced model including only eight clinical variables, ESR, u-OB, N%, IgA, CHOL, vinculin autoantibody, AST, and prolonged PT, with a LOO-CV accuracy of 92.8% (overfitting p value<0.005). As shown in **Figure 2A**, these variables have weak correlations. The mathematical formula of the model is given below:


$$sign\left(\begin{array}{c} 0.440-0.1516\times N\%+0.0845\times Vcl+B_{4}\left(ESR\right)\cdot \beta_{ESR}+B_{4}\left(u-OB\right)\cdot \beta_{u-OB}\\ +B_{4}\left(IgA\right)\cdot \beta_{IgA}+B_{4}\left(CHOL\right)\cdot \beta_{CHOL}+B_{4}\left(AST\right)\cdot \beta_{AST}+B_{4}\left(prolonged\right)\cdot \beta_{prolongedPT} \end{array}\right)$$

, with *β*_*AST*_=(3.2336,−4.3389,3.1936,1.2667)^*T*^,


$$\beta_{ESR}={\left(-0.1802,2.8125,0.8125,0.5181\right)}^{T},$$

$$\beta_{u-OB}={\left(1.9623,-2.5043,2.3149,-0.4819\right)}^{T},$$

$$\beta_{IgA}={\left(1.9071,-4.0454,2.4880,-0.5299\right)}^{T},$$

$$\beta_{CHOL}={\left(-0.8849,-2.2604,1.5635,-0.5050\right)}^{T}$$

$$\beta_{prolongedPT}={\left(-3.6318,1.8816,-1.2195,-1.9774\right)}^{T},$$

where *B*_4_(*X*) means that variable X is modeled by a 4th-order B-spline.

**Figure 4:**
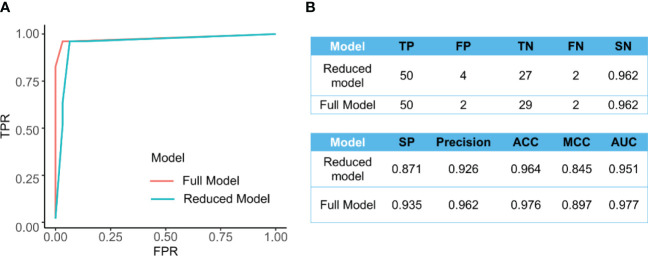
**(A)** The ROC curve for the full model (AUC=0.977) and the reduced model (AUC=0.951). **(B)** Comparison of different measurements for evaluating the performance of the full model and reduced models’ performance.

**Figure 4A** presents the ROC curves for the full and reduced models, which shows a comparative performance of these two models. In addition, **Figure 4B** shows that these two models have comparative performance in various aspects. This is consistent with the selected variables being strongly correlated, as shown in **Figure 2A**.

In the reduced model, N% and vinculin autoantibody were linearly related to SRNS. ESR, u-OB, IgA, CHOL, AST, and prolonged PT are nonlinearly related to SRNS. Only N% was negatively associated with steroid responsiveness. These biomarkers cover immune function, liver function, the urinary system, coagulation function, and other aspects, which are common organs and systems involved in INS. Hyperlipidemia and coagulation disorders are common characteristics in INS patients. Vinculin autoantibody is the only podocyte autoantibody included in the prediction model. The occurrence of vinculin autoantibodies in INS patients demonstrates the activation of B cells and damage to the podocyte actin cytoskeleton. GFB damage leads to protein leakage, and u-OB may occur. Higher vinculin autoantibody levels are less likely to be sensitive to steroids, and treatment with B-cell-depleting anti-CD20 antibodies may be helpful for these patients. Considering that these biomarkers are readily available in medical care and routine detection for INS patients, the model for predicting SRNS is easy to apply in the clinic.

Finally, we provide a user-friendly web tool for researchers to predict their results (available at https://datalinkx.shinyapps.io/srns/). Importantly, we will be delighted if others are willing to improve our model by sharing their data with us.

### Validation cohort

Due to the stronger applicability of the reduced model, 50 patients were included as validation cohort to verify the predicted effect, including 30 cases of SSNS and 20 cases of SRNS. The results showed that the reduced model obtained an accuracy of 94.0% (overfitting p value<0.005), with a sensitivity of 90.0% and a specificity of 96.7%.

## Discussion

It is well known that SSNS and SRNS have similar clinical manifestations before steroid therapy. The mechanism of steroid resistance in children with INS remains unclear. Although early genetic testing has helped clinicians formulate more personalized treatments, it does not cover all children with SRNS (22). Therefore, at the beginning of INS diagnosis, accurate prediction of steroid responsiveness is an urgent problem for clinicians. Recently, various biomarkers have been evaluated for their ability to predict different clinical phenotypes of INS (**Table 1**). Urinary proteomics is effective in predicting glomerular diseases. According to the urinary protein profile, apolipoprotein A1, urinary protein gelatinase-associated lipocalin, urine protein-bound sialic acid, urine vitamin D binding protein, and urinary protein-carbohydrate content have been regarded as new biomarkers to distinguish SSNS from SRNS (23–31). In addition to urine analysis, biomarkers in blood samples were also found to help predict the response of children with INS to steroid therapy. By flow cytometry, P-glycoprotein expression was significantly higher in SRNS (29). By ELISA, serum nephronectin concentrations were significantly lower in patients with SRNS than in patients with SSNS and controls (30). Metabolomic profiling of plasma samples from children with INS suggested that creatinine concentration, glutamine concentration, and malonate concentration were three candidate biomarkers predictive of SRNS (31). However, the number of patients recruited in the above study was very small, and only a simple univariate statistical test was conducted. Therefore, there is not enough convincing evidence to distinguish SSNS from SRNS. In this study, a total of 91 patients with INS (54 patients with SSNS, 37 patients with SRNS) were recruited, significantly exceeding the number of subjects in the above study. In addition, the usage of LOO-CV makes the training data closer to the original dataset, and there is only one sample difference between them. This has greatly filled a gap between the sample size of this study and that of other large-scale clinical studies. We comprehensively analyzed INS patients’ urine and blood samples before steroid treatment and fully extracted the disease information. To fully use this valuable dataset, we proposed a new variable selection procedure to select important variables for the response and then built SVM models for predicting SNRS. In addition, a statistical test approach is proposed for testing the overfitting of a statistical (machine learning) model, which is very important for ensuring the applicability of our model to other similar datasets. As a result, we built a full model based on all selected variables with a LOO-CV accuracy of 95.2%. To make our model more useful, we considered the correlation between variables chosen and used a stepwise forward regression method to obtain a precise model containing only eight clinical variables but with a LOO-CV accuracy of 92.6% (close to that of the full model). This is promising. Finally, we provide a user-friendly web tool to facilitate the use of our model.

**Table 1:** Biomarkers in urine and blood that distinguish between SSNS and SRNS.

Study (year)	Number of subjects	Samples	Methodology	Key findings	Ref.
Suresh CP et al. (2016)	Discovery, 15 SSNS, 12 SRNS, 5 controls. Validation, 40 SSNS, 20 SRNS, 20 controls	Urine	Proteomics (MS) and ELISA	ApoA-1 differentiated SRNS from FRNS/SDNS; alpha-2 macroglobulin, orosomucoid 2 and retinol binding protein 4 distinguished SRNS MCD from SRNS FSGS.	(20)
Kalantari S et al. (2014)	6 SSNS, 4 SRNS	Urine	Proteomics (MS)	Apolipoprotein A1 most increased in SSNS compared with SRNS; matrix remodeling-associated protein 8 decreased more in SSNS than in SRNS.	(21)
Nickavar A et al. (2016)	25 SSNS, 27 SRNS, 18 controls	Urine	Urine NGAL	Urine NGAL significantly higher in SRNS than in SSNS; optimal cutoff 0.46 ng/mg creatinine.	(22)
Gopal N et al. (2016)	47 SSNS, 23 SRNS	Urine	UPBSA	UPBSA significantly higher in SRNS than in SSNS; optimal cutoff 2.71 μg/ml of protein	(23)
Bennett MR et al. (2016)	28 SSNS, 24 SRNS, 5 controls	Urine	VDBP ELISA	Urine VDBP significantly higher in SRNS than in SSNS or controls	(24)
Gopal N et al. (2017)	47 SSNS, 23 SDNS/SRNS	Urine	Levine’s method	UPCC significantly higher in SDNS/SRNS group; threshold of 5.10 nmol/mg of protein	(25)
Badr HS et al. (2016)	20 SSNS, 16 SRNS	Blood PBMCs	Flow cytometry	P-glycoprotein expression significantly higher in SRNS.	(26)
Watany MM et al. (2018)	40 SSNS, 40 SRNS, 40 controls	Serum	NPNT ELISA	NPNT significantly higher in SSNS than in SRNS and controls, and significantly lower in SRNS than in controls.	(27)
Gooding JR et al. (2020)	30 SSNS, 15 SRNS	Plasma	Metabolomics	Metabolomic analyses from children with SSNS and SRNS identified elevated creatinine and glutamine concentrations, and reduced malonate concentrations.	(28)

ApoA-1, apolipoprotein A-1; ELISA, enzyme-linked immunosorbent assay; MS, mass spectrometry; NGAL, neutrophil gelatinase-associated lipocalin; NPNT, nephronectin; PBMC, peripheral blood mononuclear cells; SDNS, steroid-dependent nephrotic syndrome; SRNS, steroid-resistant nephrotic syndrome; SSNS, steroid-sensitive nephrotic syndrome; UPBSA, urinary protein-bound sialic acid; UPCC, urinary protein carbonyl content; VDBP, vitamin D binding protein.

method gives five clinical variables.

In a previous study, we found many kinds of podocyte autoantibodies in children with INS. The titer of these antibodies decreased with the remission of the disease. *In vivo* and *in vitro* experiments confirmed that these antibodies can cause podocyte injury and proteinuria (14). The results of the current study also found that podocyte autoantibodies helped predict responsiveness to steroid therapy and further confirmed that podocyte autoantibodies were an important part of the pathogenesis of INS. Vinculin autoantibody is the only podocyte autoantibody included in this model. There was a linear correlation between it and steroid responsiveness. This suggests that the higher the concentration of vinculin autoantibody, the more likely the children with INS will be resistant to steroids. Vinculin is a cytoplasmic protein that couples actin filaments to integrin-mediated matrix adhesion and cadherin-mediated intercellular junctions (32, 33). Vinculin is necessary to maintain the integrity of GFBs. Podocyte-specific vinculin KO mice can increase proteinuria and make the podocyte foot process disappear (34). Loss of vinculin increases FAK tyrosine phosphorylation in podocyte focal adhesions, affecting signal transduction from focal adhesions to the actin cytoskeleton. Furthermore, transfection of HEK293 embryonic kidney cells with serum- and glucocorticoid-dependent kinases significantly enhanced cell motility *via* vinculin dephosphorylation (35). Therefore, the appearance of vinculin autoantibody interferes with vinculin’s normal function and damages GFB function. The recurrence of proteinuria in children with SRNS may also be due to the repeated appearance of vinculin autoantibody. The mechanism by which vinculin autoantibody affects steroid responsiveness in children with INS needs further study *in vivo* and *in vitro*.

When the number of red blood cells in the urine reaches a certain level, the urine occult blood test results are positive. With the continuous damage of GFB, not only protein but also red blood cells can be detected in urine. In contrast, kidney damage worsens with the leakage of urinary red blood cells and proteinuria (36). This also reflects that glomerular function damage in SRNS patients is more severe than that in SSNS patients, and it is a positive feedback process.

In the clinical course of INS, a typical feature is a dysregulated coagulation state, promoted by the breakdown of the permeability barrier of the glomerular capillary wall, resulting in the leakage of high-molecular-mass proteins (37). This hypercoagulable condition is supported by several factors, such as abnormalities in platelet activation and an imbalance between anticoagulation/antithrombosis and procoagulant/prothrombotic mechanisms (38). Deep venous thrombosis of the lower extremities and renal veins are the most dangerous INS complications (39). However, based on our results, prolonged PT tends to increase in SRNS patients, which reflects dysfunction of the exogenous coagulation system. Specifically, a prolonged PT indicates an abnormal reduction in vitamin K-dependent clotting factors (II, VII, IX, X) or factor V (40). Coagulation and anticoagulation function in SRNS patients may be a complex system affected by many factors.

Hyperlipidemia is a common characteristic in INS patients. Elevated CHOL levels are largely related to an acquired LDL receptor deficiency, which limits the removal of cholesterol-rich LDL particles from circulation (41, 42). In addition, hyperlipidemia can cause accelerated ESR. Elevated ESR is also associated with increased immunoglobulin. Elevated ESR, CHOL, and IgA increase the risk of steroid resistance in INS patients. Surprisingly, increased N% is positively associated with SSNS. Acute respiratory and urinary tract infections are the most frequent triggers of relapse in SSNS patients (43). Currently, at least 50% of relapses are triggered by a viral upper respiratory tract infection, which may be linked to a nonspecific host response to infection (44).

A total of 78 variables of 87 variables collected were analyzed in the present study. After repeated attempts and optimization design ideas, we propose a full model by this new variable selection procedure with 27 important variables. To facilitate clinical application, a reduced SVM model including only eight clinical variables (ESR, u-OB, N%, IgA, CHOL, vinculin autoantibody, AST, and prolonged PT) was constructed to have a LOO-CV accuracy of 92.8%. These biomarkers cover immune function, liver function, the urinary system, coagulation function, and other aspects, which are common organs and systems involved in INS. By laboratory tests, the model comprehensively and objectively evaluates the internal conditions and disease status of INS patients, providing scientific guidance for selecting treatment methods. More importantly, the model provides a method for managing children with nonhereditary SRNS, which may solve the problem of blind medication in children with nonhereditary SRNS in the future and effectively avoid unnecessary steroid exposure.

## Conclusion

The SRNS prediction model constructed in this study comprehensively and objectively evaluated the internal conditions and disease status of INS patients, which will provide scientific guidance for selecting treatment methods for children with nonhereditary SRNS. The reason why steroids are used as first-line treatment drugs in the clinic is that compared with immunosuppressants, the effects of steroids are relatively mild, and the side effects are relatively small. In addition, most of patients are sensitive to steroids. Therefore, steroids are the first choice in the clinic. However, once predicted by our model, the possibility of steroid resistance in patients is high. Clinically, there is every reason to abandon steroids and directly choose immunosuppressants with stronger effects. These patients are likely to be ineffective after 4 weeks of steroid therapy, which not only delays 4 weeks of precious treatment time but also suffers from the side effects of steroids. At this time, it is undoubtedly a wiser decision to choose an immunosuppressant, although it has slightly larger side effects that are still controllable.

## Data availability statement

The original contributions presented in the study are included in the article/**Supplementary Material**. Further inquiries can be directed to the corresponding authors.

## Ethics statement

The Ethics Committee approved this study of the Children’s Hospital, Zhejiang University School of Medicine (2021-IRB-228). Written informed consent to participate in this study was provided by the participants’ legal guardian/next of kin. Written informed consent was obtained from the individual(s), and minor(s)’ legal guardian/next of kin, for the publication of any potentially identifiable images or data included in this article.

## Author contributions

QY and HL drafted the initial manuscript and contributed to manuscript editing. QY, YL, HL, HJ and JM collected the data from patients and contributed to manuscript editing. HJ and JM devised the conceptual ideas, contributed to the discussion and interpretation of the results, and reviewed the final manuscript. All authors contributed to the article and approved the submitted version.
