# Supplementary material for: Machine learning models for predicting steroid-resistant of nephrotic syndrome
Source: Front Immunol. 2023 Jan 26;14:1090241. doi: 10.3389/fimmu.2023.1090241 (PMC9911108; doi:10.3389/fimmu.2023.1090241)
Supplement: Supplementary file 1 [file Presentation_1.zip › Supplementary material/Supplementary Table 1.docx]

Supplementary Table 1. 87 variables of INS patients were collected in this study.

| **Clinical Variables** | | | |
| --- | --- | --- | --- |
| **Full name** | **Abbreviation** | **Full name** | **Abbreviation** |
| **Demographic characteristics** | | **Urine tests** | |
| Age | / | urine occult blood | u-OB |
| Gender | / | urine protein | UP |
| Weight | / | urine specific gravity | u-SG |
| **Hematological tests** | | urinary RBC | u-RBC |
| white blood cell counts | WBC | urinary WBC | u-WBC |
| percentage of neutrophils | N% | urinary microprotein | u-mTP |
| percentage of lymphocytes | L% | 24-hour urine protein | 24H UP |
| hemoglobin | HB | urinary microalbumin | u-mALB |
| platelet | PLT | urinary α1-microglobulin | u-α1MG |
| C-reactive protein | CRP | urinary β2-microglobulin | u-β2MG |
| erythrocyte sedimentation rate | ESR | urinary transferrin | u-TF |
| total protein | TP | urinary retinol conjugated protein | u-RBP |
| albumin | ALB | urinary IgG | u-IgG |
| globulin | GLB | uric acid | UA |
| alanine aminotransferase | ALT | 24-hour uric acid | 24H UA |
| aspartate aminotransferase | AST | urinary protein/creatinine | UP/uCr |
| serum creatinine | sCr | urinary calcium | u-Ca |
| urea | sUREA | 24-hour urinary calcium | 24H u-Ca |
| serum cystatin c | sCyC | urinary calcium/creatinine | u-Ca/uCr |
| serum β2- microglobulin | s-β2MG | urinary microalbumin/creatinine | u-mALB/uCr |
| triglyceride | TG | urinary α1-MG/creatinine | u-α1MG/uCr |
| cholesterol | CHOL | urinary β2-MG/creatinine | u-β2MG/uCr |
| antistreptococcal hemolysin O | ASO | urinary transferrin/creatinine | u-TF/uCr |
| prolonged prothrombin time | prolonged PT | urinary retinol conjugated protein/creatinine | u-RBP/uCr |
| fibrinogen | FIB | urinary IgG/creatinine | u-IgG/uCr |
| prolonged activated partial thromboplastin time | prolonged APTT | **Podocyte autoantibodies** | |
| prolonged thrombin time | prolonged TT | talin-1 autoantibody | Tln1 autoAb |
| D-dimer | / | moesin autoantibody | Msn autoAb |
| Immunoglobulin G | IgG | myosin light chain 1 autoantibody | Myh1 autoAb |
| Immunoglobulin A | IgA | vinculin autoantibody | Vcl autoAb |
| Immunoglobulin M | IgM | aconitate hydratase, mitochondrial autoantibody | Aco2 autoAb |
| C3 | / | cytoskeleton-associated protein 4 autoantibody | Ckap4 autoAb |
| C4 | / | desmoglein 1 autoantibody | Dsg1 autoAb |
| retinol conjugated protein | RBP | proteasome subunit alpha type-1 autoantibody | Psma1 autoAb |
| Interleukin-2 | IL-2 | F-actin-capping protein subunit beta autoantibody | Capzb autoAb |
| Interleukin-4 | IL-4 | filamin-A autoantibody | Flna autoAb |
| Interleukin-6 | IL-6 | plectin autoantibody | Plec autoAb |
| Interleukin-10 | IL-10 | heat shock protein HSP 90-beta autoantibody | Hs90a autoAb |
| Tumor Necrosis Factor | TNF | peptidyl-prolyl cis-trans isomerase D autoantibody | Ppid autoAb |
| Interferon-γ | IFN-γ | proxiredoxin-1 autoantibody | Prdx1 autoAb |
| CD19% | / | alpha-enolase autoantibody | Eno1 autoAb |
| CD3% | / | neuroblast differentiation-associated protein AHNAK autoantibody | Ahnak autoAb |
| CD4% | / | serine/arginine-rich splicing factor 9 autoantibody | Sfrs autoAb |
| CD8% | / | / | / |
| CD3^-^CD16^+^CD56^+^% | / | / | / |
| CD4/CD8 | / | / | / |
