# Supplementary material for: Machine learning models for predicting steroid-resistant of nephrotic syndrome
Source: Front Immunol. 2023 Jan 26;14:1090241. doi: 10.3389/fimmu.2023.1090241 (PMC9911108; doi:10.3389/fimmu.2023.1090241)
Supplement: Supplementary file 1 [file Presentation_1.zip › Supplementary material/Supplementary Table 2.docx]

**Supplementary Table 2. Summary of demographic, hematological, urinary characteristics and podocyte antibodies.**

| **Variables** | **Min.** | **1st Qu.** | **Median** | **Mean** | **3rd Qu.** | **Max.** |
| --- | --- | --- | --- | --- | --- | --- |
| Age (year) | 1.00 | 3.00 | 4.00 | 6.19 | 9.00 | 17.00 |
| Weight (kg) | 8.00 | 14.85 | 18.00 | 23.55 | 28.90 | 73.00 |
| WBC (x10^9^/l) | 4.38 | 7.56 | 9.17 | 10.39 | 12.02 | 22.40 |
| L% | 7.30 | 32.90 | 43.30 | 43.00 | 56.50 | 75.80 |
| N% | 15.10 | 34.35 | 50.40 | 48.71 | 58.25 | 88.90 |
| HB (g/l) | 70.00 | 127.00 | 133.00 | 134.00 | 141.00 | 173.00 |
| PLT (x10^9^/l) | 128.00 | 310.00 | 369.00 | 387.45 | 424.50 | 861.00 |
| CRP (mg/l) | 0.00 | 0.20 | 0.34 | 0.52 | 0.50 | 2.88 |
| ESR (mm/h) | 2.00 | 40.75 | 72.00 | 71.03 | 100.75 | 135.00 |
| u-OB | 0.00 | 0.00 | 0.50 | 0.88 | 1.00 | 3.00 |
| UP | 0.00 | 3.00 | 3.00 | 3.12 | 4.00 | 4.00 |
| u-SG (g/ml) | 1.00 | 1.02 | 1.02 | 1.02 | 1.03 | 1.05 |
| u-RBC/HP | 0.00 | 1.00 | 2.00 | 17.61 | 5.50 | 374.00 |
| u-WBC/HP | 0.00 | 1.00 | 2.00 | 6.01 | 6.00 | 42.00 |
| u-mTP (mg/l) | 197.00 | 2033.93 | 3719.90 | 5703.02 | 6368.08 | 56200.70 |
| 24H UP (mg/24H) | 142.90 | 2000.20 | 2937.40 | 4843.50 | 7086.00 | 22142.30 |
| u-mALB (mg/l) | 112.25 | 3024.48 | 6004.38 | 8163.45 | 10950.00 | 49151.60 |
| u-α1MG (mg/l) | 1.52 | 15.17 | 23.44 | 33.61 | 41.06 | 223.27 |
| u-β2MG (mg/l) | 0.04 | 0.15 | 0.24 | 0.36 | 0.37 | 3.25 |
| u-TF (mg/l) | 11.12 | 287.68 | 480.00 | 376.89 | 480.00 | 946.40 |
| u-RBP (mg/l) | 0.00 | 0.08 | 0.16 | 1.02 | 0.30 | 34.12 |
| u-IgG (mg/l) | 5.64 | 53.97 | 106.87 | 296.84 | 316.55 | 3754.60 |
| u-mALB/uCr (mg/gCr) | 138.62 | 3615.47 | 7353.18 | 8843.32 | 10033.92 | 60600.93 |
| u-α1MG/uCr (mg/gCr) | 1.21 | 21.25 | 29.78 | 37.36 | 41.85 | 176.76 |
| u-β2MG/uCr (mg/gCr) | 0.01 | 0.18 | 0.30 | 0.55 | 0.51 | 6.28 |
| u-TF/uCr (mg/gCr) | 22.71 | 261.37 | 481.31 | 525.95 | 640.03 | 2506.58 |
| u-RBP/uCr (mg/gCr) | 0.00 | 0.11 | 0.17 | 1.04 | 0.43 | 22.15 |
| u-IgG/uCr (mg/gCr) | 6.83 | 68.29 | 117.39 | 266.52 | 280.07 | 2612.28 |
| s-β2MG (ug/l) | 1301.00 | 1858.25 | 2353.00 | 2507.35 | 2666.00 | 5497.00 |
| UA (umol/l) | 965.00 | 1204.00 | 1697.00 | 1896.80 | 2134.00 | 4584.00 |
| 24H UA (umol/24H) | 93.00 | 1130.00 | 1645.00 | 1655.16 | 2126.00 | 4084.00 |
| UP/uCr ( mg/mgCr) | 0.43 | 6.04 | 10.43 | 124.43 | 16.01 | 10032.80 |
| u-Ca (mmol/l) | 0.16 | 0.64 | 0.86 | 0.94 | 1.26 | 1.79 |
| 24H u-Ca (mg/24H) | 4.30 | 22.70 | 31.40 | 35.55 | 42.90 | 159.90 |
| u-Ca/uCr | 0.01 | 0.07 | 0.10 | 0.13 | 0.16 | 0.68 |
| TP (g/l) | 31.70 | 40.45 | 45.30 | 45.62 | 49.25 | 67.90 |
| ALB (g/l) | 10.20 | 14.15 | 17.70 | 19.25 | 22.00 | 39.70 |
| GLB (g/l) | 17.90 | 24.20 | 26.10 | 26.36 | 28.90 | 37.30 |
| ALT (U/l) | 5.00 | 10.50 | 15.00 | 16.89 | 22.00 | 43.00 |
| AST (U/l) | 16.00 | 27.00 | 40.00 | 48.09 | 61.00 | 166.00 |
| sCr (umol/l) | 11.00 | 22.00 | 30.00 | 33.20 | 40.50 | 87.00 |
| sUREA (mmol/l) | 1.67 | 3.76 | 4.68 | 17.50 | 6.08 | 832.00 |
| sCyC (mg/l) | 0.43 | 0.57 | 0.67 | 4.72 | 0.80 | 364.00 |
| TG (mmol/l) | 0.65 | 1.61 | 2.43 | 3.01 | 3.83 | 12.04 |
| CHOL (mmol/l) | 3.53 | 7.81 | 10.53 | 10.90 | 13.23 | 26.61 |
| ASO (U/ml) | 0.30 | 1.40 | 2.40 | 9.14 | 6.58 | 143.40 |
| prolonged PT (s) | -11.10 | -1.95 | -1.40 | -1.43 | -0.70 | 1.20 |
| FIB ( g/l) | 0.91 | 3.99 | 4.90 | 5.02 | 5.87 | 9.40 |
| prolonged APTT (s) | -5.70 | 0.00 | 1.70 | 2.90 | 5.20 | 17.80 |
| prolonged TT (s) | -5.20 | -1.80 | -1.00 | -0.99 | 0.00 | 1.80 |
| D-dimer (mg/l) | 0.09 | 0.36 | 0.78 | 1.09 | 1.39 | 4.88 |
| IgG (g/l) | 0.80 | 1.90 | 2.80 | 3.72 | 5.13 | 10.80 |
| IgA (g/l) | 0.08 | 0.85 | 1.10 | 1.41 | 1.62 | 3.76 |
| IgM (g/l) | 0.39 | 1.13 | 1.58 | 1.69 | 2.07 | 3.67 |
| C3 (g/l) | 0.80 | 1.13 | 1.32 | 1.31 | 1.46 | 2.26 |
| C4 (g/l) | 0.03 | 0.25 | 0.35 | 0.37 | 0.48 | 0.76 |
| RBP (mg/l) | 13.00 | 22.50 | 27.80 | 30.73 | 35.55 | 90.30 |
| total IgE (IU/ml) | 17.80 | 81.35 | 311.00 | 446.17 | 782.50 | 1200.00 |
| IL-2 (pg/ml) | 1.90 | 2.60 | 2.80 | 2.84 | 3.10 | 3.70 |
| IL-4 (pg/ml) | 1.10 | 2.20 | 2.40 | 2.42 | 2.80 | 3.80 |
| IL-6 (pg/ml) | 3.60 | 6.00 | 8.10 | 83.72 | 49.20 | 703.50 |
| IL-10 (pg/ml) | 1.70 | 3.10 | 4.10 | 6.04 | 5.10 | 26.90 |
| TNF (pg/ml) | 1.00 | 1.40 | 1.90 | 3.49 | 2.30 | 42.00 |
| IFN-γ (pg/ml) | 1.00 | 1.30 | 2.10 | 2.43 | 2.90 | 6.50 |
| CD19% | 0.00 | 8.88 | 12.75 | 13.61 | 17.18 | 34.90 |
| CD3% | 54.20 | 67.20 | 75.40 | 73.63 | 80.45 | 88.20 |
| CD4% | 13.00 | 32.35 | 37.80 | 38.55 | 45.25 | 58.80 |
| CD8% | 11.10 | 23.15 | 28.20 | 28.21 | 33.45 | 54.30 |
| CD3-CD16+CD56+% | 1.90 | 5.13 | 7.50 | 8.49 | 11.30 | 20.70 |
| CD4/CD8 | 0.24 | 1.03 | 1.46 | 2.56 | 1.79 | 58.60 |
| Tln1 autoAb | 0.00 | 23.40 | 39.10 | 47.72 | 58.65 | 370.70 |
| Msn autoAb | 0.00 | 10.20 | 20.70 | 24.16 | 35.35 | 137.60 |
| Myh1 autoAb | 0.00 | 21.85 | 36.20 | 39.14 | 55.20 | 136.30 |
| Vcl autoAb | 0.00 | 24.30 | 44.30 | 61.22 | 71.45 | 371.30 |
| Aco2 autoAb | 0.00 | 17.45 | 30.90 | 39.85 | 50.05 | 371.00 |
| Ckap4 autoAb | 0.00 | 14.70 | 27.50 | 32.05 | 45.00 | 144.50 |
| Dsg1 autoAb | 0.00 | 9.90 | 22.20 | 26.84 | 39.25 | 255.60 |
| Psma1 autoAb | 0.00 | 16.75 | 36.80 | 49.55 | 57.75 | 870.30 |
| Capzb autoAb | 0.00 | 27.55 | 54.30 | 58.90 | 83.70 | 363.40 |
| Flna autoAb | 0.00 | 11.80 | 31.10 | 39.76 | 49.60 | 440.90 |
| Plec autoAb | 0.00 | 0.55 | 18.00 | 191.54 | 31.15 | 15656.20 |
| Hs90a autoAb | 0.00 | 13.30 | 25.60 | 35.65 | 41.50 | 574.40 |
| Ppid autoAb | 0.00 | 11.45 | 23.70 | 31.42 | 47.25 | 179.70 |
| Prdx1 autoAb | 0.00 | 11.70 | 20.60 | 35.40 | 34.10 | 989.50 |
| Eno1 autoAb | 0.00 | 21.40 | 32.70 | 42.58 | 49.70 | 425.00 |
| Ahnak autoAb | 0.00 | 16.05 | 31.00 | 53.96 | 51.35 | 1675.40 |
| Sfrs autoAb | 0.00 | 13.90 | 27.10 | 37.24 | 45.45 | 614.80 |
